# Frequency of cancer in children residing in Mexico City and treated in the hospitals of the Instituto Mexicano del Seguro Social (1996–2001)

**DOI:** 10.1186/1471-2407-4-50

**Published:** 2004-08-13

**Authors:** Servando Juárez-Ocaña, Guadalupe González-Miranda, Juan Manuel Mejía-Aranguré, Mario Enrique Rendón-Macías, María del Carmen Martínez-García, Arturo Fajardo-Gutiérrez

**Affiliations:** 1 Unidad de Investigación Médica en Epidemiología Clínica, Hospital de Pediatría, Centro Médico Nacional Siglo XXI, Instituto Mexicano del Seguro Social, México; 2 Registro de Cáncer en Niños, Hospital de Pediatría, Centro Médico Nacional Siglo XXI, Instituto Mexicano del Seguro Social, México; 3 División de Promoción de Desarrollo, Coordinación de Investigación en Salud del Instituto Mexicano del Seguro Social, México

**Keywords:** Epidemiology, cancer in children, diagnostic stage, Hodgkin's disease, neuroblastomas

## Abstract

**Background:**

The objective of this article is to present the frequency of cancer in Mexican children who were treated in the hospitals of the Instituto Mexicano del Seguro Social in Mexico City (IMSS-MC) in the period 1996–2001.

**Methods:**

The Registry of Cancer in Children, started in 1996 in the IMSS-MC, is an on-going, prospective register. The data from 1996 through 2001 were analyzed and the different types of cancer were grouped according to the International Classification for Cancer in Children (ICCC). From this analysis, the general and specific frequencies by age and by sex were obtained for the different groups of neoplasms. Also, the frequency of the stage of the disease that had been diagnosed in cases of children with solid tumors was obtained.

**Results:**

A total of 1,702 new cases of children with cancer were registered, with the male/female ratio at 1.1/1. Leukemias had the highest frequency with 784 cases (46.1%) and, of these, acute lymphoblastic leukemias were the most prevalent with 614 cases (78.3%). Thereafter, in descending order of frequency, were tumors of the central nervous system (CNST) with 197 cases (11.6%), lymphomas with 194 cases (11.4%), germinal cell tumors with 110 cases (6.5%), and bone tumors with 97 cases (5.7%). The highest frequency of cancer was found in the group of one to four year-olds that had 627 cases (36.8%). In all the age groups, leukemias were the most frequent. In the present work, the frequency of Hodgkin's disease (~4%) was found to be lower than that (~10%) in previous studies and the frequency of tumors of the sympathetic nervous system was low (2.3%). Of those cases of solid tumors for which the stage of the disease had been determined, 66.9% were diagnosed as being Stage III or IV.

**Conclusions:**

The principal cancers in the children treated in the IMSS-MC were leukemias, CNST, and lymphomas, consistent with those reported by developed countries. A 2.5-fold reduction in the frequency of Hodgkin's disease was found. Of the children, the stage of whose disease had been determined, two thirds were diagnosed as having advanced stages of the disease.

## Background

The frequency of malignant neoplasms in children has been found to vary among countries. For example, in children in Canada, the United States, and Europe, the three most common cancers are leukemias, tumors of the central nervous system (CNST), and lymphomas [1-3], whereas in children in Latin America, the order of frequencies is distinct: leukemias are still in first place, with lymphomas being more common than are CNST [2,4-6]. In other countries such as Nigeria, Malawi, and Egypt, lymphomas are the principal neoplasias [2].

The percentage of cases of each type of neoplasm in relation to the total number of cancers is also different. In the developed countries, the percentages for leukemias range between 30 and 37%; for CNST, between 18 and 27%; and for lymphomas, between 7 and 12% [1-3]. In Latin America, the percentages for leukemias are between 27 and 44%; of lymphomas, between 13 and 22%; and of CNST, between 10 and 19% [2,4-6]. In African countries, the percentages of lymphomas range between 30 and 64% [2]. In Asiatic countries such as Japan and China, the percentages of leukemias have been found to be between 30 and 40%; of CNST, between 12 and 20%; and of lymphomas, between 10 and 20% [2].

The study of the frequency of cancer in children not only is of interest to the clinical physician because it helps him/her to establish the pre-testing probability for a child suspected of having cancer [7], but also is of interest to the personnel in charge of the planning and programming of the medical attention for these children, as pertains to the assignment of human resources (physicians, specialized nurses, social workers, and others) and to the allotment of financial resources (centers providing medical attention, laboratories, imaging facilities, medicines, etc.) that are necessary for treating the children [8].

In Mexico, there existed data only from retrospective studies on the epidemiology of cancer in children and those studies had been carried out prior to 1993 [9,10]. Therefore, it was necessary to use more recent data, especially those from prospective studies in which the under-reporting of cases was reduced.

The objective of this paper is to present the frequency of malignant neoplasias in the child population residing in the area served by the Instituto Mexicano del Seguro Social (IMSS-MC). The data were obtained from the Registry of Cancer in Children which was started in 1996 at, and is maintained by the Hospital de Pediatría del Centro Médico Nacional "Siglo XXI" of the Instituto Mexicano del Seguro Social in Mexico City (IMSS-MC).

## Methods

### Type of study

Observational, descriptive, and prospective hospital inquiry.

### Population studied

The Instituto Mexicano del Seguro Social (IMSS) offers medical attention to the population of workers and their families, which comprises 50% of the Mexican population [10]. The IMSS divides the population that it attends into four regions: North, South, East, and West. The IMSS-Mexico City (IMSS-MC) attends the population of the Southern region, which includes the populations in various states in the country, in addition to that of Mexico City. With the objective of avoiding a bias in the selection of the population for the present study, we included for analysis only cases from those states in which we were sure that more than 90% of the cases presented had been registered. This would be true of cases from the states that are located closest to Mexico City and of the cases in which the children who developed cancer had to be sent to Mexico City for treatment. Therefore, the cases analyzed came from Mexico City and from the following selected states: State of Mexico, Morelos, Guerrero, and Chiapas. It should be mentioned that not only the cost of treatment, but also the cost of transportation to Mexico City is covered by IMSS. This financial support provided by IMSS to families helps to ensure that cases of cancer come to the attention of IMSS-MC and are duly registered.

Newly diagnosed cases of malignant neoplasias in children less than 15 years of age treated in the IMSS-MC were included in this study. In 100% of these cases, the diagnosis was confirmed by histological tests and/or by aspiration of the bone marrow.

### Participating facilities

The IMSS-MC has two hospitals that provide medical attention to children with cancer. The Departments of Hematology and of Pediatric Oncology of both the Hospital de Pediatría of the Centro Médico Nacional "Siglo XXI" (HP) and the Hospital General del Centro Médico Nacional "La Raza" (HR) participated in this study. Both facilities have the infrastructure (competent personnel and suitable technology) needed for the precise diagnosis of a cancer.

### Study period

Included in this study were the cases attended from 1 January 1996 to 31 December 2001.

### Study variables

Prior to carrying out the study, a form for recording the variables of interest was designed. For this article, the variables analyzed were the following: type of neoplasia, sex of the patient, age at the time of diagnosis, and the stage of the cancer for those children with solid tumors.

### Procedure

At each hospital, a fulltime nurse was assigned to register all new cases of cancer. Prior to collecting the data, each nurse was instructed in procedure necessary for obtaining all the different variable of the study. The nurse interviewed the parents and reviewed the clinical record of each child in order to obtain all the necessary information.

The nurse was also taught how to encode and to determine the stage of cases of solid tumors. The standardization of the coding and determination of the stage of the solid tumors was done by all personnel (three physicians and two nurses) concerned with the registry; an excellent concordance was obtained (unweighted Kappa of 0.85) [11].

In addition to other duties related to the Registry, each nurse spent three days a week in the oncology and hematology departments of the hospital (HP or HR), searching for cases of children who, being suspected of having cancer, had been registered in a specific file and boarded at the hospital. After reviewing the clinical record in each file, the nurse either included the case in the study (encoding the data if the diagnosis of cancer had been confirmed) or eliminated the case if the diagnosis was not confirmed. If for any reason, the patient was discharged from the hospital and the diagnosis was undetermined, the nurse reviewed the clinical record in the clinical archive of the hospital in order to learn what the final diagnosis was.

To encode the different cases of cancer, topographical and morphological coding was used. The second edition of the "International Classification of Diseases for Oncology" (ICD-O-2) was used for the cases collected from 1996 through 1999; the third edition (ICD-O-3), for the cases collected from 2000 through 2001 [12,13]. For determining the stage of cases of lymphomas and carcinomas, the recommendations of the American Joint Committee on Cancer (AJCC) and the International Union Against Cancer (IUAC) were used [14]. The stages for tumors of the central nervous system (CNST), neuroblastoma, retinoblastoma, renal tumors (Wilms' tumor), and those of the liver, bones, soft tissues, and germinal cells (GCT) were determined following the recommendations of the Children's Oncology Group [15].

The Child-Check Program developed by the International Agency for Research on Cancer (IARC) [16] was used to evaluate the internal consistency of the individual registries of cancer and to convert the nomenclature of ICD-O-2 to the International Classification of Childhood Cancer (ICCC) [17]. This program made crosses between different variables in order to find inconsistencies among the collected data. The crosses that were made were sex-topography, sex-histology, age-tumor type, unlikely combinations of topography-morphology, errors between date of birth and diagnosis, and duplication of cases. The result was a list of combinations, although either improbable or of low probability, that were needed for review in order to verify data or to correct data by rechecking the records of the patients. Cases of the ICD-O-3 that were not included in the Child-Check Program were classified by using other procedures and the data entered manually.

### Statistical analysis

Cases were grouped according to the ICCC [17] that has established 12 different groups of cancer in children. From these were calculated the absolute and relative frequencies, both in general and according to sex and to age, the latter category being divided into four subgroups: under one year; one to four year-olds; five to nine year-olds; and ten to 14 year-olds. Due to the fact that the procedure for the determination of the stage of the disease was initiated in 1 Jun 1998, such determinations were made in 658 of the case of solid tumors. Therefore, the frequencies of the diagnostic states were based on this number of cases of children with solid tumors.

## Results

A total of 1,702 cases of malignant neoplasias were analyzed. Of these, in the order of the most frequently found, were the following types of tumors: leukemias, 784 cases (46.1%); CNST, 197 cases (11.6%); lymphomas, 194 cases (11.4%); germinal cell tumors (GCT), 110 cases (6.5%); and bone tumors (BT), 97 cases (5.7%); and the remainder of the neoplasias were found in low percentages (Table 1).

Table 1 shows that, in examining the subtypes of the different groups of neoplasias, it was found that, of the leukemias, the most frequent were the acute lymphoblastic leukemia (n = 614; 78.3%); of the lymphomas, the non-Hodgkin lymphomas [Burkitt, non Burkitt, and nonspecific together (n = 129; 66.5%)]; of the CNST, the astrocytomas (n = 97; 49.2%); of the tumors of the sympathetic nervous system (SNST), neuroblastomas and ganglioneuroblastomas together (n = 36; 92.3%); of the renal tumors, nephroblastoma [Wilms' tumor (n = 62; 87.3%)]; of hepatic tumors, hepatoblastoma (n = 26; 86.7%); of BT, osteosarcoma (n = 70; 72.2%); of the sarcomas of the soft tissues, rabdomyosarcoma and embryonic sarcoma together (n = 49; 55.1%); of the GCT, gonadal tumors (n = 80; 72.7%); and of the carcinomas, adrenocortical, malignant melanoma and skin carcinoma together (n = 9; 50.1%).

The percentages of the different neoplasias showed variations according to sex and age group. These findings modified the pattern of presentation and made it different from the overall pattern. In males, over 70% of the cases consisted of the following types of tumors: leukemias (n = 412; 46.6%); lymphomas (n = 138; 15.6%); and CNST (n = 84; 9.5%). In females, 73.6% of the cases consisted of leukemias (n = 372; 45.5%); CNST (n = 113; 13.8%); GCT (n = 61; 7.5%); and lymphomas (n = 56; 6.8%). Overall, the ratio of males to females was 1.1; however, this ratio varied for the different groups of neoplasias, most notably a high of 2.5 for lymphomas and a low of 0.8 for both hepatic tumors, BT and GCT (Table 2).

Table 2 shows that, for all age groups, leukemias had the highest frequency, ranging between 27.9 to 50.5%. In second and third place for the different age groups were the following types of tumors: under one year of age, retinoblastoma (n = 15; 17.4%) and GCT (n = 13; 15.1%); 1–4 year-olds, CNST (n = 69; 11.0%) and retinoblastoma (n = 56; 8.9%); 5–9 year-olds, lymphomas (n = 79; 16.2%) and CNST (n = 59; 12.1%); and 10–14 year-olds, lymphomas (n = 66; 13.2%) and BT (n = 65; 12.9%).

With respect to the neoplasias in patients from other states in the Mexican Republic, leukemias were also found to have the highest frequency, followed by lymphomas and/or CNST, with discrete variations in the frequencies as mentioned above. It should be noted that in one state (Chiapas), although retinoblastoma was only the fourth most frequent there, its frequency (8.9%) was one of the highest (Table 3).

In regard to the spread of the disease at the time of diagnosis for those children the stage of whose solid tumors had been determined, 89 (13.5%) were Stage I; 129 (19.6%), Stage II; 242 (36.8%), Stage III; and 198 (30.1%), Stage IV or higher.

## Discussion

This is the first report of data, covering the six year period from 1996–2001, taken from the on-going Registry of Cancer in Children that was started in 1996 in Mexico City by the Instituto Mexicano del Seguro Social (IMSS-MC). The strategy of having placed a nurse in each of the two hospitals involved with the Registry of Children with Cancer resulted in the great majority (more than 90%) of the new cases that were treated by the two hospitals being identified and duly registered. We therefore concluded that this Registry of Cancer in Children was one of the most complete of its kind undertaken in Mexico City.

Having access to the clinical records of the patients served to improve the quality of the data that was registered because, since cases were registered as soon as they were diagnosed, few cases were overlooked.

The quality of the data was also enhanced by the use of the Child-Check Program, with which the possible errors, not only of registry but also of capture, were reviewed and were eliminated upon rechecking the respective clinical record in the hospital files. As mentioned in Methods, in 100% of the cases, the histopathology reports for children with solid tumors, or the reports on the aspirated bone marrow for children with leukemia, were obtained. Therefore, the data that was obtained for the children with cancer who were residents in the area covered by IMSS-MC and treated in Mexico City were the most precise that have been gathered to date in Mexico.

The frequency of a disease in a population is a method of obtaining the pre-test probability that a patient has before a diagnostic test is performed [7]. It is important that this value be known, because there is a direct correlation with the positive predictive value of the test. This, in turn, is the probability that an individual whose test result was positive has of having the suspected disease [7]. That is, in the case of the children who were attended in the tertiary health care hospitals in IMSS-MC, the pre-test probability for a child whose parents sought medical attention (without taking into account his/her symptomatology) is a 43.4% chance of having some form of leukemia, an 11.1% chance of lymphoma, or a 12.6% chance of CNST. This probability increases or decreases depending on the symptomatology that the child presents, on the test requested, and on the result (positive or negative) of said test. Knowing the frequency of diseases in general and, in this case, the frequency of the different types of cancer that Mexican children present is therefore an important aid in diagnosis. The same can be said for the frequency of cancer by age and by sex.

As has been mentioned, knowing the frequency of diseases serves in the estimation of administrative needs, not only with respect to personnel but also to the equipment and supplies necessary for diagnosis and treatment, and for providing medical attention in general and in particular for children with cancer [8]. Given that, from the data, 46.1% of these children develop leukemia, appropriate provision has to be made for their treatment, as well as for the children with other forms of cancer.

A more clinical aspect, particular to children with cancer, that indicated the spread of their disease was the stage of the disease at the time of diagnosis. It was established that 66.9% were in stages III or IV, a finding that generally means the prognosis for the patient is not good. This datum was consistent with, and more precise than the result of the previously reported retrospective study, in which it was found that 56.4% had been diagnosed as having an advanced stage (III or IV) of the disease [10]. This fact indicates that, for Mexican children that develop a cancer, programs for integrated medical attention must be designed such that early diagnosis is a priority. Although the early diagnosis of cancer in children as a factor in a good prognosis is controversial [18], it is probable that such a program would have a great impact in Mexico. For this reason, as has been pointed out in prior studies [19], the influence of various factors such as the patient's family (educational level, socio-economic status, etc.), the type of cancer, the age of the child, the health system, and the physicians that care for the child on intake must be taken into account.

Although the results obtained for the different groups of cancer in Mexican children in this study showed general agreement with data previously reported, there were some differences. Consistent with data obtained in other studies [9,10], it was found that leukemia, CNST, and lymphoma were the principal neoplasias and that Mexican children have one of the highest percentages of leukemias in the world (46.1% vs. 27–44%) [2,20,21].

In contrast to previous studies in Mexico, the frequency of Hodgkin's disease was notably lower, thus reducing the overall frequency of lymphoma to slightly lower than that of CNST and putting it in the range reported for developed countries (11.4% vs. 7–12%) [2]. We consider this reduction in the frequency of Hodgkin's disease a noteworthy and probably real effect, not just an artifact of the quality of the registry.

The previous studies, being retrospective, had a greater tendency to under-register the number of cases and therefore result in an lower frequency than the actual value; yet, here, with a more accurate study, the frequency obtained was lower still (10% a 3.8%) (Table 4) [9,10]. However, we consider it necessary to obtain the incidence rates to confirm this and will do so for a forthcoming study in which we will present exclusively the incidence of cancer in children who are residents of Mexico City. It should be mentioned that the same phenomenon (reduction in the HD) was shown by Linet et al. [22] in their study of children in the U.S. during the period 1974 to 1995. There has been no explanation of this phenomenon because no program attempting to reduce HD in Mexico or in other parts of the world has been established. However, should an infectious agent be one of the causes in the development of HD, it is possible that the indiscriminant use of antiviral and/or antibiotics in Mexico may play a role. Perhaps future studies will be able to explain the phenomenon.

In this and the two prior studies in Mexico, the frequency of CNST was found to be less than that in developed countries (18–27%). However, the lowered frequency of lymphomas found in the present study changed the hierarchical pattern of tumors, with CNST now having just edged out lymphomas for second place. We do not consider that this population has, strictly speaking, the pattern of U.S.-Canada-Europe, nor that of Latin America. We can say that the pattern of neoplasias in Mexican children was found to be a phenomenon in transition. It was interesting that the pattern of presentation found in children in Mexico City was different to that of the children that live in the northern part of the country (data not shown). The pattern in children from the north was similar to that of children in the U.S. and Canada, in that the principal neoplasias were leukemias, CNST, and lymphomas and had very similar frequencies [23,24]. This finding suggested the possibility that factors which cause cancer in children in the north of Mexico may be different to those causing cancer in children in Mexico City.

Data for children under one year of age showed that, whereas neuroblastoma was the principal tumor for this age group in developed countries [1,3], in our study it was leukemias (27.9%), with the frequency of neuroblastoma being much lower than in developed countries (10.5 vs. 27.4). This finding is one that should be followed up because there are two factors that would have affect the reported number of cases and, hence the frequency: Because Mexico does not have the screening programs for detecting children with neuroblastoma that other countries do [25], it was probable that cases of this disease were not being diagnosed. Also, it is known that some of these tumors do regress spontaneously [26].

Another interesting aspect of the present study was that one of the highest frequencies of GCT (6.5%) in the world was found, a frequency similar to that for some Asiatic countries, such as Japan and Singapore (6.8%) [2]. We do not have an explanation of this finding, but studies directed toward establishing the causes of this high frequency should be carried out.

Finally, it should be mentioned that one state of the Mexican Republic, Chiapas, was found to have one of the highest frequencies of retinoblastoma (8.9%), a frequency very similar to that of countries of Africa (Zimbabwe, 9.6%) and in India (Madras; 9.4%) [2]. Chiapas is one of the poorest states in Mexico and, as has been suggested, it is possible that the state of nutrition may play a role in the development of this tumor [27]. However, as for Hodgkin's Disease, it will be necessary to calculate the incidence rates to make this observation more precise.

## Conclusions

It may be concluded that, in the children residing in Mexico City that were included in this study, the principal neoplasias were leukemias, CNST, and lymphomas, findings that were consistent with previously published data. It was found that, in comparison to previous studies in Mexico City, there was a reduction in the frequency of lymphomas and especially of Hodgkin's disease, and that, of the children with solid tumors, two thirds were diagnosed as having advanced stages (III-IV) of the disease.

## Competing interest

None declared.

## Authors' contributions

S J-O analyzed the data and wrote the first draft of the manuscript. G G-M registered, recorded, and analyzed the data. JM M-A, ME R-M, and MC M-G conceived and the designed the study and analyzed the data. A F-G conceived and designed the study, analyzed the data, and provided guidance to all aspects of this project. All authors read and approved the final manuscript.

## Pre-publication history

The pre-publication history for this paper can be accessed here:



## Figures and Tables

**Table 1:** Frequency of cancer in children served by the Instituto Mexicano del Seguro Social and treated in hospitals in Mexico City (1996–2001).

**Diagnostic group**	**Total frequency**		**Frequency by group**	
	**n**	**%**	**n**	**%**
**I. Leukemias**	**784**	**46.1** 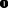	**784**	**100.0**
IA. Acute lymphoblastic leukemia	614	36.1	614	78.3
IB. Acute non-lymphoblastic leukemia	146	8.6	146	18.6
CI. Chronic myeloid leukemia	16	1.0	16	2.0
ID. Other specific leukemias	4	0.2	4	0.5
IE. Nonspecific leukemias	4	0.2	4	0.5
II. Lymphomas*	**194**	**11.4** 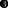	**194**	**100.0**
IIA. Hodgkin disease	65	3.8	65	33.5
IIB. Non-Hodgkin lymphoma	104	6.1	104	53.6
IIC. Burkitt lymphoma	21	1.2	21	10.8
IIE. Nonspecific lymphomas	4	0.2	4	2.1
**III. Central Nervous System Tumors**	**197**	**11.6** 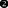	**197**	**100.0**
IIIA. Ependymoma	29	1.7	29	14.7
IIIB. Astrocytoma	97	5.7	97	49.2
IIIC. Primitive neuroectodermal tumors	52	3.1	52	26.4
IIID. Other gliomas	9	0.5	9	4.6
IIIE. Other specific intracranial and intraspinal neoplasms	9	0.5	9	4.6
IIIF. Other nonspecific intracranial and intraspinal neoplasms	1	0.1	1	0.5
**IV. Symphatetic Nervous System Tumors**	**39**	**2.3**	**39**	**100.0**
IVA1. Ganglioneuroblastoma	6	0.3	6	15.4
IVA2. Neuroblastoma	30	1.7	30	76.9
IVB1. Meduloepitelioma	1	0.1	1	2.6
IVB3. Neuroepithelioma	1	0.1	1	2.6
IVB4. Olfactory neuroblastoma	1	0.1	1	2.6
**V. Retinoblastoma**	**73**	**4.3**	**73**	**100.0**
V1. Retinoblastoma	46	2.7	46	63.0
V2. Differentiated retinoblastoma	24	1.4	24	32.9
V3. Undifferentiated retinoblastoma	3	0.2	3	4.1
**VI. Renal Tumors**	**71**	**4.2**	**71**	**100.0**
VIA1. Nephroblastoma	62	3.6	62	87.3
VIA2. Rabdoid sarcoma	2	0.2	2	2.8
VIA3. Clear-cell sarcoma	6	0.3	6	8.5
VIB. Renal Carcinoma	1	0.1	1	1.4
**VII. Hepatic Tumors**	**30**	**1.8**	**30**	**100.0**
VIIA. Hepatoblastoma	26	1.5	26	86.7
VIIB1. Hepatic carcinoma	1	0.1	1	3.3
VIIB2. Hepatic carcinoma undifferentiated	1	0.1	1	3.3
VIIB3. Neuroendocrine carcinoma	1	0.1	1	3.3
VIIB4. Acinar adenocarcinoma	1	0.1	1	3.3
**VIII. Bone Tumors**	**97**	**5.7** 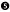	**97**	**100.0**
VIIIA. Osteosarcoma	70	4.1	70	72.2
VIIIB. Chondrosarcoma	4	0.2	4	4.1
VIIIC. Ewing sarcoma	18	1.1	18	18.6
VIIID. Other specific malignant tumors	3	0.2	3	3.1
VIIIE. Unspecified malignant bone tumors	2	0.2	2	2.1
**IX. Soft-Tissue Sarcomas**	**89**	**5.2**	**89**	**100.0**
IXA. Rhabdomyosarcoma and embryonal sarcoma	49	2.9	49	55.1
IXB. Fibrosarcoma, neurofibrosarcoma and others fibromatous neoplasms	21	1.2	21	23.6
IXD. Other specific soft tissue sarcomas	17	1.0	17	19.1
IXE. Unspecified soft tissue sarcomas	2	0.2	2	2.2
**X. Germ Cell Tumors**	**110**	**6.5** 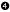	**110**	**100.0**
XA. Intracranial and intraspinal germ cell tumors	15	0.9	15	13.6
XB. Other and unspecified non-gonadal germ cell tumors	13	0.8	13	11.8
XC. Gonadal germ cell tumors	80	4.7	80	72.7
XD. Gonadal carcinomas	2	0.2	2	1.8
**XI. Carcinomas**	**18**	**1.1**	**18**	**100.0**
XIA. Adrenocortical carcinoma	3	0.2	3	16.7
XIB. Thyroid carcinoma	1	0.1	1	5.5
XIC. Nasopharyngeal carcinoma	1	0.1	1	5.5
XID. Malignant melanoma	3	0.2	3	16.7
XIE. Skin carcinoma	3	0.2	3	16.7
XIF. Other and unspecified carcinomas	7	0.4	7	38.9
**Total**	**1702**	**100.0**	**1702**	**100.0**

* 40 cases of Histiocytosis were not included Note: Numbers in circles indicate the five highest frequencies, in descending order

**Table 2:** Frequency of cancer, according to sex and to age group, in children served by the Instituto Mexicano del Seguro Social and treated in hospitals in Mexico City (1996–2001).

**Diagnostic group**	**Age group (years)**								Sex				
	**< 1**		**1–4**		**5–9**		**10–14**		**Male**		**Female**		**Ratio**
	**N**	**%**	**n**	**%**	**n**	**%**	**n**	**%**	**n**	**%**	**n**	**%**	**M/F**
I. Leukemias	24	27.9 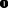	301	48.0 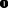	246	50.5 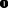	213	42.4 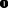	412	46.6 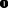	372	45.5 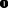	1.1
II. Lymphomas*	0	0.0	49	7.8	79	16.2 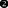	66	13.2 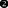	138	15.6 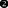	56	6.8	2.5
Hodgkin disease	0	0.0	7	1.1	26	5.3	32	6.4	43	4.9	22	2.7	1.9
Non-Hodgkin lymphoma	0	0.0	42	6.7	53	10.9	34	6.8	95	10.7	34	4.1	2.8
III. CNST	6	7.0	69	11.0 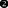	59	12.1 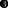	63	12.5	84	9.5 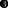	113	13.8 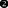	0.7
IV. SNST	9	10.5	21	3.3	5	1.0	4	0.8	20	2.3	19	2.3	1.1
V. Retinoblastoma	15	17.4 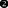	56	8.9 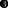	1	0.2	1	0.2	34	3.8	39	4.8	0.9
VI. Renal Tumors	7	8.1	44	7.0	15	3.1	5	1.0	39	4.4	32	3.9	1.2
VII. Hepatic Tumors	7	8.1	11	1.8	7	1.4	5	1.0	13	1.5	17	2.1	0.8
VIII. Bone Tumors	0	0.0	6	1.0	26	5.3	65	12.9 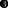	43	4.9	54	6.6	0.8
IX. Soft Tissue Sarcomas	5	5.8	29	4.6	23	4.7	32	6.4	43	4.9	46	5.6	0.9
X. Germ Cell Tumors	13	15.1 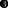	38	6.1	19	3.9	40	8.0	49	5.5	61	7.5 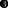	0.8
XI. Carcinomas	0	0.0	3	0.5	7	1.4	8	1.6	9	1.0	9	1.1	1.0
Total	86	100	627	100	487	100	502	100	884	100	818	100	1.1

* 40 cases of Histiocytosis were not included M: male; F: female Numbers in circles indicate the three highest frequencies, in descending order CNST: central nervous system tumors SNST: sympathetic nervous system tumors

**Table 3:** Frequency of cancer in children* of different states of the mexican republic served by the Instituto Mexicano del Seguro Social and treated in hospitals in Mexico City (1996–2001).

**Diagnostic group**	**Chiapas**		**Mexico City**		**Guerrero**		**State of Mexico**		**Morelos**	
	**n**	**%**	**n**	**%**	**n**	**%**	**n**	**%**	**n**	**%**
I. Leukemias	46	37.4 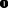	280	43.5 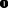	33	31.7 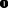	377	51.5 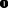	48	48.0 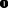
II. Lymphomas**	13	10.6 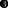	83	12.9 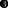	20	19.2 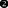	67	9.2 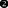	11	11.0 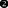
Hodgkin disease	4	3.3	21	3.3	6	5.8	32	4.4	2	2.0
Non-Hodgkin lymphoma	9	7.3	62	9.6	14	13.5	35	4.8	9	9.0
III. CNST	18	14.6 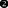	85	13.2 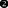	19	18.3 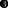	67	9.2 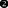	8	8.0 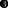
IV. SNST	1	0.8	17	2.6	7	6.7 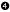	12	1.6	2	2.0
V. Retinoblastoma	11	8.9 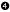	22	3.4	5	4.8	31	4.2	4	4.0
VI. Renal tumors	4	3.3	26	4.0	5	4.8	30	4.1	6	6.0
VII. Hepatic tumors	3	2.4	18	2.8	0	0.0	8	1.1	1	1.0
VIII. Bone tumors	9	7.3 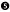	31	4.8	7	6.7 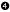	43	5.9 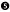	7	7.0 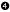
IX. Soft tissue sarcomas	8	6.5	35	5.4 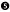	4	3.8	36	4.9	6	6.0 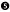
X. Germ cell tumors	6	4.9	42	6.5 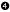	2	1.9	55	7.5 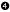	5	5.0
XI. Carcinomas	4	3.3	4	0.6	2	1.9	6	0.8	2	2.0
Total	123	100	643	100	104	100	732	100	100	100

Note: Numbers in circles indicate the five highest frequencies in descending order *Children 0–14 years **40 cases of histiocytosis were not included CNST: central nervous system tumors SNST: sympathetic nervous system tumor

**Table 4:** Comparison of the frequency of cancer in children* in Mexico City and in four selected countries

**Diagnostic Group**	**Mexico City 1980–1991^9/^**		**Mexico City 1992–1993^10/^**		**Mexico City 1996–2001**		**USA-SEER White 1983–1992^2/^**		**German Federal Republic 1985–1990^2/^**		**France 1983–1992^3/^**		**Cuba 1986–1990^5/^**	
	**n**	**%**	**n**	**%**	**n**	**%**	**n**	**%**	**n**	**%**	**n**	**%**	**n**	**%**
I. Leukemias	1706	34.9 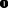	78	39.2 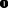	784	46.1 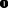	1757	30.7 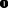	2422	34.4 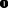	746	29.4 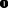	454	30.7 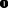
II. Lymphomas**	888	18.2 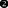	34	17.6 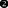	194	11.4 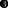	616	10.7 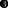	781	11.1 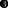	310	12.2 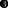	271	18.3 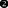
Hodgkin disease	524	10.7	18	9.0	65	3.8	259	4.5	279	4.0	103	4.1	84	5.7
Non-Hodgkin lymphoma	364	7.5	16	8.6	129	7.6	357	6.2	502	7.1	207	8.1	187	12.6
III. CNST	496	10.2 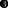	25	12.6 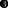	197	11.6 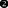	1222	21.4 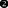	1392	19.8 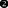	538	21.2 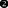	223	15.1 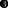
IV. SNST	133	2.7	6	3.0	39	2.3	469	8.2 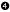	552	7.8 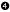	244	9.6 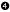	104	7.0 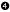
V. Retinoblastoma	420	8.6 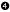	5	2.5	73	4.3	172	3.0	208	2.9	61	2.4	40	2.7
VI. Renal Tumors	279	5.7	9	4.5	71	4.2	366	6.4	464	6.6	152	5.9	69	4.7
VII. Hepatic Tumors	70	1.4	1	0.5	30	1.8	83	1.5	82	1.2	23	0.9	21	1.4
VIII. Bone Tumors	321	6.6 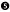	9	4.5	97	5.7 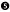	267	4.7	359	5.1	129	5.1	87	5.9
IX. Soft Tissue Sarcomas	238	4.9	10	5.0 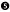	89	5.2	387	6.8 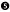	492	7.0 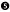	154	6.1 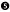	91	6.2 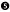
X. Germ Cell Tumors	256	5.2	17	8.6 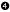	110	6.5 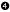	165	2.9	222	3.2	71	2.8	34	2.3
XI. Carcinomas	55	1.1	3	1.5	18	1.1	193	3.4	55	0.8	108	4.3	55	3.7
XII. Nonspecific	18	0.4	0	0	0	0.0	21	0.4	7	0.1	2	0.1	29	2.0
Total	4880	100	198	100	1702	100	5718	100	7036	100	2538	100	1478	100

Note: Numbers in circles indicate the five highest frequencies, in descending order *Children 0–14 years **40 cases of histiocytosis were not included CNST: central nervous system tumors SNST: sympathetic nervous system tumor References 2,3,5,9,10.
